# Genetic screening and response to drug therapy in familial hypercholesterolemia

**DOI:** 10.3389/fmolb.2026.1784302

**Published:** 2026-04-24

**Authors:** Yuzhi Lu, Qianwen Chen, Wenjuan Zhang, Jiangtao Dong, Lingfeng Zha

**Affiliations:** 1 Department of Cardiology, Union Hospital, Tongji Medical College, Huazhong University of Science and Technology, Wuhan, China; 2 Hubei Key Laboratory of Biological Targeted Therapy, Union Hospital, Tongji Medical College, Huazhong University of Science and Technology, Wuhan, China; 3 Hubei Provincial Engineering Research Center of Immunological Diagnosis and Therapy for Cardiovascular Diseases, Union Hospital, Tongji Medical College, Huazhong University of Science and Technology, Wuhan, China; 4 Department of Pediatric Cardiology, Maternal and Child Health Hospital of Hubei Province, Tongji Medical College, Huazhong University of Science and Technology, Wuhan, China; 5 Department of Geriatrics, The Central Hospital of Wuhan, Tongji Medical College, Huazhong University of Science and Technology, Wuhan, China; 6 Department of Cardiovascular Surgery, The Central Hospital of Wuhan, Tongji Medical College, Huazhong University of Science and Technology, Wuhan, China

**Keywords:** familial hypercholesterolemia, genetic testing, LDLR, pcsk9, whole exome sequencing

## Abstract

**Objectives:**

Familial hypercholesterolemia (FH) is an autosomal genetic disorder, which is significantly underdiagnosed. Here, we aimed to identify the genetic causes of a FH family and clarify the clinical diagnosis of the patient and then provide personalized treatment plan.

**Materials and Methods:**

We recruited a three-generation Chinese family with a history of FH and conducted genetic testing by Whole exome sequencing and Sanger sequencing. The potential effect of mutation identified was predicted using various software such as SIFT, Polyphen-2, Mutation Taster, RNAfold, AlphaFold and the conservation was tested using multiple sequence alignments by ClustalX. The pathogenicity of the identified mutation was evaluated according to the 2015 ACMG/AMP Standards and Guidelines.

**Results:**

A frame shift insertion mutation (c.2517_2518insCA, p. C839fs) in *LDLR* was identified in proband, which showed to have a deleterious effect and has not been reported before. According to the 2015 ACMG/AMP, this nonsense pathogenic mutation was classified as “pathogenic”. This LOF mutation was thought to influence RNA stability by changing the free energy dynamics of the RNA molecule, while it also changes protein structure by removing an essential LDLR functional domain. With personalized drug treatment, such as statins, ezetimibe, and PCSK9 inhibitor, the serum lipids of the proband were well controlled and the prognosis was good. The further cascade screening of family members identified three carriers of the mutation, who need close follow-up and regular monitoring of lipid changes to prevent future cardiovascular events.

**Conclusion:**

Here, we identified a novel mutation in *LDLR* (c.2517_2518insCA, p. C839fs) for FH. Hyperlipidemia patients carrying this mutation will respond favorably to statins and PCSK9 inhibitor. The finding expanded our understanding of Phenotype–Genotype Correlations of FH with *LDLR* gene mutations and emphasized the important role of genetic testing and genetic screening in the diagnosis and intervention of FH.

## Introduction

Familial hypercholesterolemia (FH) is a prevalent autosomal genetic disorder characterized by premature coronary artery disease (CAD), corneal arcus, and tendon xanthomas which are caused by elevated levels of circulating low-density lipoprotein cholesterol (LDL-C) (Reiner, 2015; Chen et al., 2019). According to epidemiological studies, the overall prevalence of FH ranges from 1 in 200 to 1 in 250 worldwide, with China accounting for roughly 8% of all FH patients worldwide. However, due to poor public awareness of FH and insufficient genetic testing, the diagnostic rate of FH patients is very limited and remains less than 1% in China, posing a major health burden (Chen et al., 2019; Wilemon et al., 2020). Despite increased knowledge of FH, diagnosis and treatment remain woefully inadequate, with only 10% of the FH population being identified and treated effectively. In recent years, FH has risen to prominence as a major worldwide public health concern that has drawn considerable international attention. The FH Foundation and the World Heart Federation gathered FH specialists from 40 countries in 2018 to demand increased awareness of FH and the treatment of severe FH as a global health concern (Wilemon et al., 2020).

Based on the results of its genetic variation, this inherited metabolic illness may be divided into two types: heterozygous FH (HeFH) and homozygous FH (HoFH). HeFH is a genetic autosomal dominant or codominant disease that affects 0.2% of the world population. HeFH patients have plasma LDL-C levels that are 2–3 times greater than usual (Sjouke et al., 2015). HoFH is associated with more severe symptoms than HeFH, despite the lower frequency. Approximately one case out of every 160,000 to 300,000 people is affected by HoFH. The plasma LDL-C levels of HoFH patients are 6–8 times higher than that of normal subjects as a result of the gene dose effect (Li et al., 2016). Furthermore, in rare circumstances, HoFH might be identified as a recessive trait (Cuchel et al., 2014).

The genetics of FH are extremely complex. 1. Genetic heterogeneity. FH is caused by pathogenic mutations in genes involved in LDL metabolism in the liver mediated by the low density lipoprotein receptor (LDLR). The most prevalent mutations are in the genes encoding LDLR, apolipoprotein B (*APOB*), proprotein convertase subtilin/kexin type 9 (*PCSK9*), and LDL receptor adaptor protein 1 (*LDLRAP1*). A pathogenic variant in those genes could be detected in 80% of patients with clinically diagnosed FH (Cuchel and McGowan, 2021). Among them, *LDLR* gene variants are the most common, with more than 2,000 *LDLR* variants reported so far. Recently, genes encoding signal-transducing adaptor family member 1 (*STAP1*), epoxide hydrolase 2 (*EPHX2*), growth hormone receptor (*GHR*), and apolipoprotein E (*APOE*) have also been reported to be related to FH (Di Taranto et al., 2020). 2. Variant clusters. Several FH-causative mutations are more likely to occur in specific geographic locations due to founder effects. 3. Phenotypic variability. The clinical profile of HeFH patients, including LDL-C values, is diverse. HoFH patients show similar complexity (Cuchel et al., 2014). 4. Polygenic FH. Polygenic FH is found in around 20% of people with clinically confirmed FH. Small alterations in lipid metabolism proteins occur as a result of the accumulation of prevalent LDL-C raising variations. Interestingly, monogenic FH individuals have a greater cardiovascular risk than polygenic FH patients. 5. Phytosterolemia. Phytosteroidemia is an autosomal recessive condition characterized by increased cholesterol and phytosterol absorption in the intestine. Sometimes, it is indistinguishable from real FH. Given the complexities of FH, diagnosing and treating this disease becomes extremely challenging.

The major clinical symptoms of FH include dramatically elevated blood LDL-C levels and early-onset atherosclerotic cardiovascular disease (ASCVD), which might be asymptomatic in the early stages. Undiagnosed HoFH patients can develop severe early-onset ASCVD, and many die before the age of 20. Patients with undiagnosed heterozygous FH appear signs or symptoms of ASCVD (e.g., angina pectoris) or ASCVD adverse events (e.g., myocardial infarction [MI], sudden cardiac death) in early middle age. Coronary artery calcification, one of the characteristics of CAD, is seen in HeFH patients aged 11–23 years. Tendon xanthomas are most commonly found in the Achilles tendon and back of the hand, although they can appear elsewhere. The corneal arcus is a white or gray ring-shaped area that appears at the edge of the cornea. Finally, some patients may also have aortic stenosis. FH patients have a 20-fold increased risk of ASCVD than the general population, and untreated individuals have exceptionally high cardiovascular mortality. Early diagnosis of FH is severely lacking, particularly in China, and most FH patients are recognized only after the development of CAD, missing out on early therapy. As a result, early screening and diagnosis are critical measures for lowering the risk of ASCVD development and improving clinical prognosis in FH patients.

In this study, we present a case of a 48-year-old man with massive increases in circulating LDL-C values, and his CTA revealed atherosclerosis and three-vessel coronary artery calcification. We performed Whole exome sequencing (WES) for the proband since it is an efficient and cost-effective approach for genetic testing. WES coupled with Sanger sequencing found a unique heterozygous frameshift indel mutation in *LDLR* (c.2517_2518insCA, p. C839fs) as the pathogenic explanation via multiple biological analysis. According to the 2015 ACMG/AMP, this nonsense pathogenic mutation was classified as “pathogenic”. This LOF mutation was thought to influence RNA stability by changing the free energy dynamics of the RNA molecule, while it also changes protein structure by removing an essential LDLR functional domain. We combined the genetic test results, clinical phenotype and drug response to develop a personalized medication regimen for him. With personalized drug treatment, such as statins, ezetimibe, and PCSK9 inhibitor, the serum lipids of the proband were well controlled and the prognosis was good. The further cascade screening of family members identified three carriers of the mutation, who need close follow-up and regular monitoring of lipid changes to prevent future cardiovascular events.

## Materials and methods

### Objects of study

We identified the three-generation Chinese family with a history of FH according to the Dutch Lipid Clinic Network (DLCN) criteria. DLCN criteria for scoring FH are based on cholesterol concentrations, physical examination, family history and molecular genetic tests. Patients with DLCN scores greater than 8 are diagnosed with definite FH. In our study, the proband is a 48-year-old male, previously hospitalized in our department due to elevated LDL-C values discovered during a health screening. All affected family members underwent detailed physical examination and medical history. Other clinical data of the proband included age, sex, routine blood test which included fasting lipid profiles (LDL-C, HDL-C, Triglyceride and Total Cholesterol), blood glucose, complete blood counts, liver and kidney function tests, imaging results such as two-dimensional, Doppler, and tissue Doppler echocardiography, multi-slice computed tomography coronary angiography (coronary CTA) and laboratory tests, such as quantitation of light chain in blood and urine, quantitation of serum autoantibodies and total immunoglobulin, and serum AFP level. Then we re-examined their clinical data, described their family pedigree and recruited other family members.

### DNA preparation

The proband (II-2) and five of his family members (II-1, III-1, III-2, III-3 and III-4) each had 1.5 mL of peripheral blood drawn (in the EDTA vacuum container). DNA was extracted following the instruction of the DNeasy Blood &Tissue Kit (Cat#69506, QIAGEN, GmBH, Germany). The concentration and purity of the DNA samples were tested by NanoDrop2000 spectrophotometer, the average DNA concentration is 60 ng/μL, OD260/OD280 = 1.8. The purified DNA with a starting concentration of 2 ng/μL was tested by Qubit®2.0 Fluorometer and 1% agarose gel electrophoresis.

### Whole exome sequencing

The DNA sample of the proband was provided to do genetic testing by WES. For the sample to be sequenced, individual library preparations, hybridizations, and captures were performed following the protocol of SureSelectXT Target Enrichment System for Illumina Paired-End Sequencing Library (Agilent Technologies, Inc. 5,301 Stevens Creek Rd Santa Clara, CA 95051 United States of America). Assess quantity of library with Qubit® 2.0 Fluoromete. Use 2,100 Bioanalyzer High Sensitivity DNA Assay to assess the quality and size range as instructed in the reagent kit guide. TruSeq PE Cluster Kit (Illumina) was used for cluster generation in an Illumina cBOT instrument following the manufacturer’s protocol (cBotTM User Guide). Libraries were loaded into each lane of flow cell. Sequencing was performed on an Illumina HiSeq X instrument (Illumina) by the manufacturer’s protocol (HiSeq® X SystemUser Guide). Multiplexed paired-reads run were carried out with 125 cycles. Then, to identify the pathogenic variant, the proband’s sequencing data were analyzed and annotated. Single-nucleotide variants (SNVs) and indels were defined with SAMtools, while copy number variations (CNVs) were defined by CoNIFER, referring to the human reference genome (hg19). Allele frequency was achieved by comparing each variant with numbers of public databases, for instance, Genome Aggregation Database (gnomAD), Exome Aggregation Consortium (ExAC), Exome Sequencing Project (ESP), and 1000 Genomes. Several popular prediction tools: SIFT, PolyPhen-2, MutationTaster, and GERP++ were used to evaluate the potential influence on the protein function. Then the known pathogenic variants were detected with the help of the Clinvar database.

According to the variant annotations, we use the following prioritization strategies to identify candidate variants related to the phenotypes: (1) variants, not in the exonic and splicing regions are excluded; (2) variants with minor allele frequency (MAF) > 0.01 are excluded; (3) synonymous variants are excluded; (4) non-conservative variants are excluded (GERP++conservation prediction score ≤2); (5) variants that are not damaging through the protein function prediction including SIFT, Polyphen2, MutationTaster are excluded.

### Sanger sequencing

To confirm the potential FH causative variant identified by WES, we amplified the genomic DNA fragments by PCR with a 25 µL volume PCR reaction system including 12.5 μL of 2 × TSINGKE Master Mix, 2 μL DNA, 9.5 μL Ultrapure water, 0.5 μL of each primer (forward 5′-ATGGTACGATGCCCGTGTTT-3′ and reverse 5′-CTGAATGAGCGCACAGAAGC-3′) in the proband and his familial members, and then carried out Sanger sequencing. Variant segregation in this family was determined based on the genotype and state of each family member.

### Genetic risk score (GRS) analysis

The current study showed that only 40%–80% of persons with DLCN scores >8 have a single gene etiology. In individuals with clinical FH but no variations known to cause FH, polygenic etiology should be taken into consideration since they have a higher number of common raised lipid variants. In order to evaluate the proband’s polygenic contribution to FH, we calculated the GRS by making use of the data from an exome-wide association study, which analyzed the effects of 345 lipid-associated variants on LDL-C levels in >300,000 individuals.

### Functional prediction analysis of pathogenic mutation

Firstly, multi-sequence alignment across species indicated the conservation of the area where the mutation occurred. The conservative nature of amino acid position reflects its importance. Negative consequences are more probable to appear when a mutation occurs in a protein’s key functional or structural area.

Secondly, we used the RNA secondary structure prediction program (RNAfold web server, http://rna.tbi.univie.ac.at/cgi-bin/RNAWebSuite/RNAfold.cgi) to assess the influence of mutations on RNA secondary structure. Free energy is the energy necessary to modify the structure in this prediction program. As a result, the structure is more stable if the matching value is less. RNAfold receives native and wild-type RNA sequences in FASTA format and applies the Mccaskill algorithm to produce a string that represents the secondary structure of the RNA and a folded mountain plot that illustrates the energy differences between native and mutant sequences. We compared mutant and wild type minimal free energy (MFE) by importing mutant and wild sequences, respectively.

Finally, we evaluated the effect of mutation on protein structure through the AlphaFold Protein Structure Database (https://www.alphafold.ebi.ac.uk/), which can make a confident prediction of amino acid structural positions in the human proteome (Tunyasuvunakool et al., 2021). AlphaFold can reveal the per-residue confidence score (pLDDT) of amino acid structural positions, and it’s currently thought that low pLDDT implies that the amino acid is extremely likely to be unstructured in isolation, the higher the pLDDT, the better the match between the AlphaFold prediction and the experimental structure. We also acquired the resolved structure from Protein Data Bank since a portion of the LDLR protein (3M0C: chain C,1-715aa) had been crystallized. Then, we respectively modeled the remaining structural sections by a combinatorial method, including comparison modeling, threading, and *ab initio* modeling using the I-Tasser website (George Priya Doss et al., 2013; Thirumal Kumar et al., 2018). Based on the confidence score, we chose the best model. A mix of template-based assignment and profile-based prediction is used to predict the B-factor profile (BFP). B-factor is a measure of the degree of the natural thermal mobility of residues and atoms in proteins. This value is calculated by threading template proteins from the PDB together with the sequence profiles obtained from sequence databases in I-TASSER. According to the distribution and prediction of the BFP, residues with BFP values greater than 0 are less stable in experimental structures. Simultaneously, we utilized the RoseTTAFold (https://robetta.bakerlab.org/) prediction tool to compare two structures using the Rosetta-constrained energy-minimization technique (Yang et al., 2020). Chimera software (1.11.2) was used to offer the visualization of the proteins.

### Clinical interpretation of pathogenic mutation

American College of Medical Genetics/Association for Molecular Pathology (ACMG/AMP) guidelines are used to describe the causality of variants found in Mendelian disease-associated genes and classify variants into five categories based on different data sources: pathogenic, likely pathogenic, uncertain significance, likely benign, and benign. The pathogenicity assessment and the clinical interpretation of the identified mutation in this study were on the basis of the 2015 ACMG/AMP standards Guidelines (Richards et al., 2015).

## Results

### Case presentation

**FIGURE 1:**
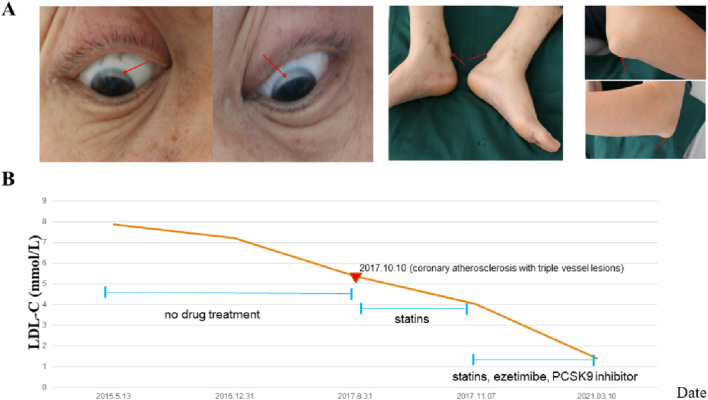
Clinical symptoms of the proband. **(A)** Arcus senilis and tenoxanthoma of the proband. **(B)** LDL-C changes of the proband during recent 6 years.

**FIGURE 2:**
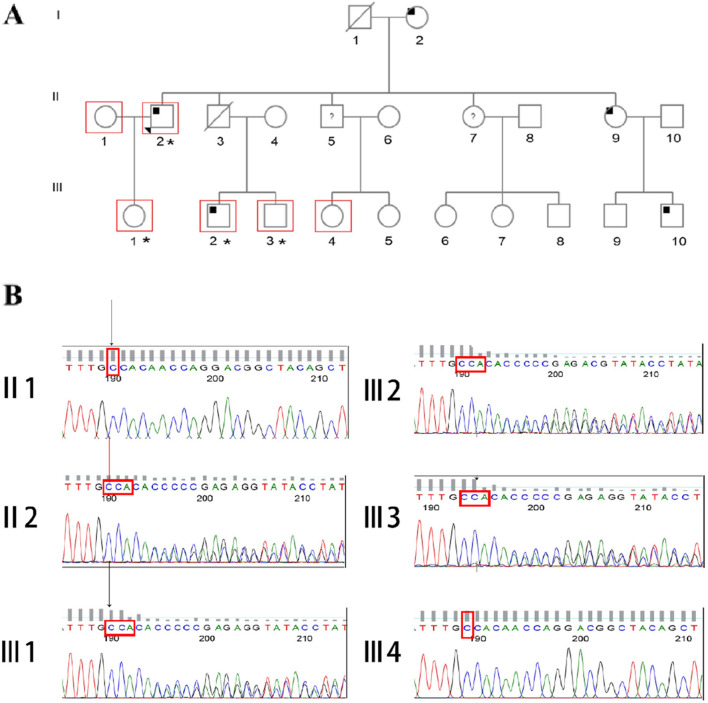
Pedigree and sanger sequencing chromatograms of c 2517_2518insCA in *LDLR*. **(A)** Family pedigree. Black arrow represents proband; asterisk indicates mutation carrier; the red box represents those who have undergone genetic screening. **(B)** The sanger sequencing of c.2517_2518insCA, arrow indicates mutation site.

During a health screening, the proband, a 48-year-old man was admitted to our department with elevated LDL-C results. He was clinically diagnosed with FH at the age of 44 years old, prior to which the patient underwent three medical examinations and started lipid management only after the third medical examination. According to his own statements, proband’s mother, one of the proband’s siblings, and two of the proband’s nephews all had this illness, but the rest of his family was well (Table 1). They are all Han people from Hubei, China, and have no history of genetic illness in their families. This proband was an occasional smoker, had low blood pressure (blood pressure 97/67 mmHg), and a body mass index of 24.7 kg/m^2^. The earliest physical examination reports from other groups suggested mixed hyperlipidemia, hyperuricemia, and mild fatty liver, and his medical records showed his LDL-C level spread from 5.36 to 7.86 mmol/L without treatment. His LDL-C level was lowered to 4.03 mmol/L after a month of aspirin and atorvastatin treatment. Computed tomography angiography (CTA) and Color Doppler Ultrasonography revealed coronary artery atherosclerosis, three-vessel coronary artery calcification, and plaque development in bilateral carotid and lower extremity artery. During our physical examination, tendon xanthomas and corneal arcus were also discovered. We classified him as FH using the DLCN standard since his DLCN score was up to 16. Pedigree based analysis of the proband suggested the positive family history of FH and they are inherited in an autosomal dominant pattern. The biochemical findings of his mother (I-2), one sister (II-9) and two nephews (III-2, III-10) were consistent with an FH diagnosis. His mother suffered from multiple MI and his brother died in a car accident with no way of knowing his lipid profile. Figure 1 depicts his clinical symptoms and drug use, whereas Figure 2A depicts his pedigree.

**TABLE 1:** Clinical and biochemical characteristics of FH families.

Member	Genotype	TC (mmol/L)	TG (mmol/L)	HDL (mmol/L)	LDL (mmol/L)	Clinical phenotype
I-2	-	9.30	1.50	1.85	6.3	Hyperlipidemia
II-1	C	-	-	-	-	Normal/oral
II-2	CCA	9.90	3.94	0.94	7.86	FH
II-5	-	6.20	1.00	2.09	3.50	Normal
II-7	-	6.40	1.80	1.82	3.78	Dyslipidemia
II-9	-	9.60	2.40	1.71	6.02	Hyperlipidemia
III-1	CCA	-	-	-	-	Normal/oral
III-2	CCA	5.99	0.57	1.23	4.71	Hyperlipidemia
III-3	CCA	-	-	-	-	Normal/oral
III-4	C	3.87	1.06	1.19	2.27	Normal
III-10	-	-	-	-	-	Hyperlipidemia/oral

### Genetic identification of pathogenic mutation

In order to search for the determinant variants leading to FH, we performed the WES on the proband. The proportion of on-target reads in all samples of the project was about 74%, the coverage was over 99%, and the mean depth was about 120×. Good capture efficiency provided enough data. Based on the aligned reads, we identified 97,779 initial variants (SNVs, indels). This data was screened five times according to its frequency, pathogenicity, etc. Then we filtered the variants associated with the phenotype “FH”. Out of the filtered candidate variants, we noted a frameshift insertion mutation (c.2517_2518insCA) in *LDLR,* which is the most known FH-causing gene. The insertion was a new mutation and therefore was not presented in public databases, including GnomAD, ExAC, ESP, and 1,000 Genomes. Besides, due to belonging to the insertion mutation, prediction of possible protein functions was not achieved, including SIFT, Polychen2, and Mutation Taster. The mutation was located in exon 17 of the *LDLR* gene, within the cytoplasmic domain, which is positioned on chromosome 19 q12. This insertional mutation causes a frameshift in the *LDLR* gene’s coding sequence and the substitution of the native amino acid asparagine with a variant threonine at position 840, resulting in an extension of the following polypeptide chain.

To confirm the veracity of the WES result and to further test his family for this mutation, we used Sanger sequencing, which revealed that this mutation was present in the proband and his daughter (III-1) as well as two nephews (III-2 and III-3), but not in his wife or niece (II-1 and III-4) (Figure 2B). This FH familial mutation was verified to have an autosomal dominant inheritance pattern by integrating family history with genetic screening findings. The bearers of the mutation are all heterozygous.

### Genetic risk score for LDL-C of the proband

The GRS was derived using the genetic score for LDL-C to determine the proband’s polygenetic vulnerability. Since the advent of a meta-analysis to identify genetic loci associated with FH in 2010, a variety of multigene scoring models have emerged. The concept of polygenic hypercholesterolaemia was introduced in 2013 by Talmud et al. who created a GRS model containing 12 single nucleotide polymorphisms (SNPs), in which 6 SNPs are central. In contrast, none of the 12 SNPs mentioned above were present in the proband. In Ontario, researchers also developed a 10-SNP GRS in which individuals with an extreme weighted GRS greater than 1.96 were classified as having a higher risk of polygenic hypercholesterolaemia. Again, this was not observed in the proband. Based on the collective impact of the various variations, the available Iceland evidence shows that a 1-unit increase in the genetic score approximates an increase of one SD in LDL-C levels and a rise of 1.04 mmol/L per 1-unit increase in the genetic score. We discovered 46 common LDL-C-related SNPs in the proband’s WES data, which had previously been reported in large-scale GWfdtr5AS investigations. Depending on the proband’s genotypes and the impact sizes of these SNPs from the GWAS data, the proband’s weighted genetic score for those 46 SNPs is −0.05119. The combined effect of several genes is modest as compared to monogenic FH because the GRS is lower than the national average for big groups. In other words, the cumulative impact of common variations on FH, as measured by the generated polygenic risk score, is minor. Hence, the monogenic mutation may still be the primary cause of FH in the proband.

### Functional analysis of pathogenic mutation

**FIGURE 3:**
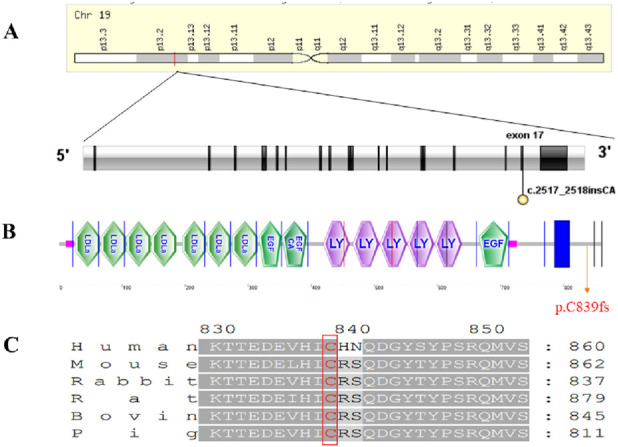
The location and conservation of the mutation (c.2517_2518insCA, p. C839fs). **(A)** The location of the mutation on the *LDLR* gene. **(B)** LDLR protein domain and mutation location. **(C)** Conservation of the mutation location.

We investigated the functional implications of this recently found mutation, which is responsible for the pathophysiology of FH. According to conservative predictions, this mutation was found in a gene area with strong evolutionary conservation among several species such as Gorilla, Panicus, Pig, Rabbit, Rat, and others (Figure 3). Two bases (CA) were added between the 2,517 and 2,518 positions of the LDLR cDNA, causing a frame-shift in LDLR transcripts and protein sequence changes from amino acid 839 and a longer polypeptide chain (p.C839fs) (Figure 3). The LDLR protein has a total of 860 amino acids, with the intracellular domain accounting for 839 to 860 locations. Intracellular domains are highly conserved and regulate endocytosis and intracellular transport, which is critical for LDLR activity.

**FIGURE 4:**
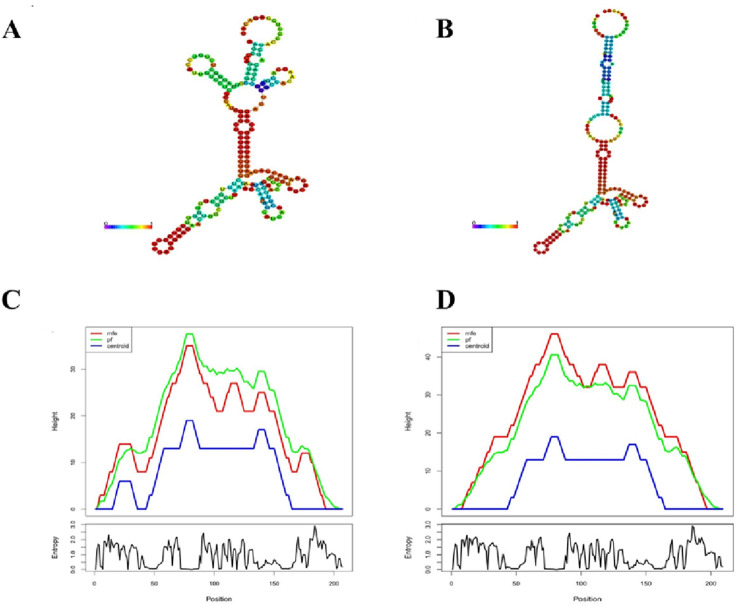
Human *LDLR* RNA secondary structure prediction by RNAfold. **(A,B)** RNA secondary structures of *LDLR* predicted for wild-type and mutant, minimal free energy (MFE) is showed by color gradient between 0 and 1. **(C,D)** Mountain plot (MP) exhibition of MFE, thermodynamic ensemble (pf) and the centroid structure prediction for wild-type and mutant RNA. MP shows the secondary structure in height vs. position, where the helices are showed in slopes, loops in plateaus and hairpin loops in the peaks. The figure below is the predicted entropy value of RNA structure. The higher the entropy value, the lower the previous stability of RNA structure.

The mutation’s computational impact on RNA secondary structure was then evaluated. The relative stability of mutant LDLR RNA secondary structure is relatively poor, with −29.1 kcal/mol compared to wild-type reporters (MEF was −37.3 kcal/mol), according to MEF calculations of LDLR centroid structure. As a result, mRNAs folding pattern, tertiary structure, and function are likely to be affected by the more unstable RNA with c.2517_2518insCA mutation (Figure 4).

**FIGURE 5:**
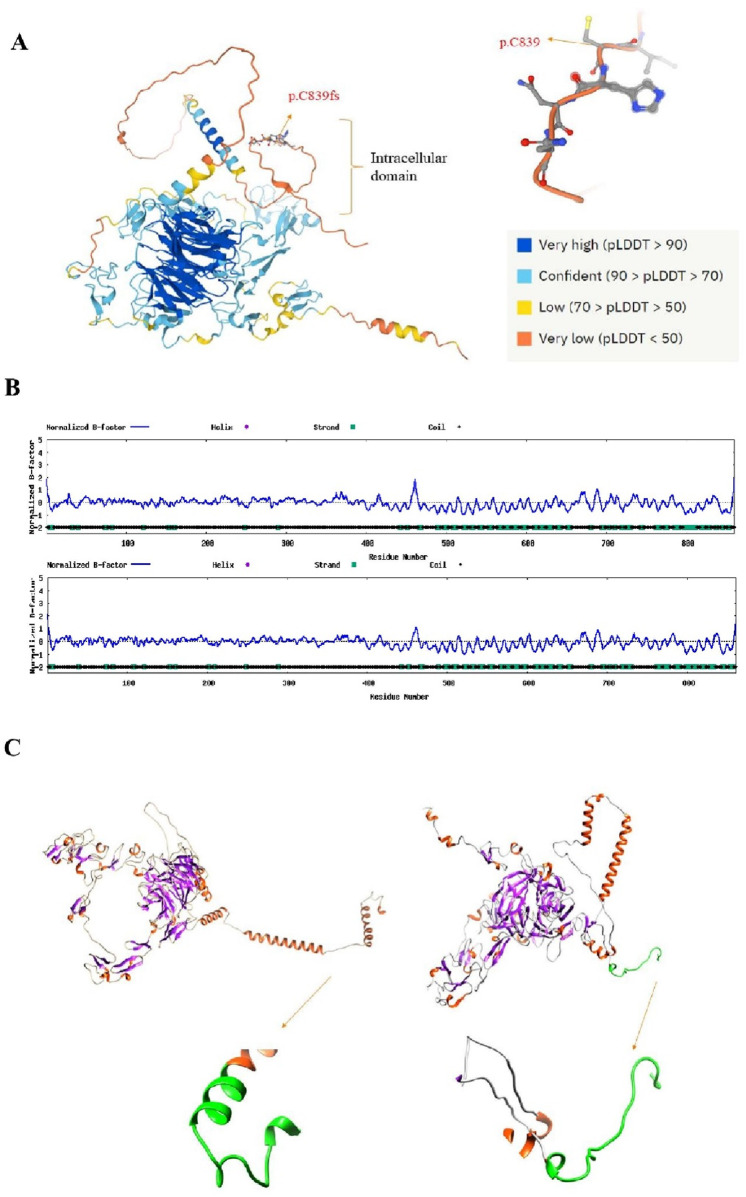
Human LDLR protein structure prediction by AlphaFold. **(A)** Predicted structure of Human LDLR protein. The different colors represent the per-residue confidence score (pLDDT) of each amino acid, pLDDT is between 0 and 100. **(B)** Predicted mutant and wild type LDLR secondary structure from I-Tasser. **(C)** The mutant and wild LDLR protein structure visualizations according to the results of RoseTTFold.

Model confidence from AlphaFold revealed that the pLDDT is very low for the position of 839 amino acids (pLDDT = 24.7), and the overall pLDDT for the position of 839–860 amino acids is also very low (the average pLDDT 50) (Figure 5). The current rationale for low pLDDT areas found by AlphaFold is that they are likely localized in unstructured regions. Long sections with pLDDT <50 in the current dataset have a distinct ribbon-like look and should not be taken as structures but rather as a prediction of disorder. The ligand-binding domain (292aa), EGF precursor homology approx. (400aa), O-linked sugars (58aa), transmembrane domain (22aa), and cytoplasmic domain (50aa) are all known domains of the LDLR protein. However, because the crystal structure of LDLR has not yet been fully resolved, we used the I-Tasser confidence score to estimate the secondary structure of LDLR. In comparison to the wild type, protein secondary structure and BFP had been significantly changed, and the frameshift protein possessed a newly produced C-terminal peptides that are critical for the protein’s stability and function (Figure 5). Furthermore, according to RoseTTAFold’s findings, the secondary structure of mutant LDLR protein had also been significantly altered (Figure 5). In conclusion, it is estimated that the function of mutant LDLR cannot be sustained.

### Clinical interpretation of the mutation

The nonsense pathogenic mutation (c.2517_2518insCA, p. C839fs) of *LRLR* was classed as “pathogenic” according to the 2015 ACMG/AMP because it was a null mutation inside the coding region of the *LDLR* gene, and loss of function (LOF) is a well-known mechanism causing FH (Chora et al., 2022). This interpretation is supported by other evidence, which is summarised in Table 2.

**TABLE 2:** Clinical interpretation of the mutation by ACMG/AMP 2015 guideline.

Mutation	Mutation type	Mutation classification	Criteria	Strength of criteria
c.2517_2518insCA	Frameshift mutation	Pathogenic	PVS1	Very strong
			PM4	Moderate
			PM2	Moderate
			PP1	Support
			PP3	Support
			PP4	Support

## Discussion

In this study, we reported a case of FH and detailed his clinical characteristics in-depth. We detected a novel heterozygous frameshift harmful mutation (c.2517_2518insCA, p. C839fs) in *LDLR* using a genetic test and found three carriers of the mutation in this family by a cascade of screening. Inherited *LDLR* mutations greatly reduce or eliminate LDLR function, as does this mutation, which causes abnormal amino acids at the C-terminus starting from amino acid 839, leading to a complete change in the intracellular domain of LDLR protein, which is confirmed to be associated with endocytic transport of LDLR. A literature search and database search revealed a total of 1,915 *LDLR* mutations associated with FH, of which 46% are missense mutations and 18% are frameshift mutations (Chora et al., 2022). The initial few amino acids in the intracellular domain are encoded by exon 17, with the majority of the domain encoded by exon 18. A total of 49 FH-related mutations in exon 17 and 5 in exon 18 have been previously reported. Frameshift mutations led to the degradation of proteins due to translation errors, thereby reducing LDLR protein levels and subsequently interfering with receptor assembly in those individuals carrying the mutation. Free energy-based RNA stability studies predict that this mutation destabilized the structure of mRNA and ultimately affected its folding pattern. Free energy alterations also affected the structural stability of LDLR protein. We therefore predicted that identified mutation in *LDLR* is recognized as the cause in thecontext of FH.

Elevated cholesterol level is a common risk factor for CAD, and therefore individuals with FH are more likely to develop CAD early. A meta-analysis of 26 RCTs, including up to 170,000 respondents, indicated that lowering LDL-C by 1 mmol/L reduced cardiovascular risk by roughly 22%. Recent research has also established the notion of “cumulative LDL-C burden”, which suggests that a healthy 55-year-old with a cumulative LDL-C level of 160 mmol is at high risk of developing CAD. If no therapy is given, HeFH individuals in their 35s can develop CAD; however, if medication is given from the ages of 18 or 10, CAD development can be delayed until age 48 or 53. Patients with HoFH who do not receive treatment may develop CAD by the age of 12.5 years (Nordestgaard et al., 2013). In addition, the prevalence of FH is very high in adults under the age of 50 who had MI. FH was found in 10% of the young MI population, 20% of the young MI population with a family history of premature CAD, 40% of the young MI population with LDL >4.14 mmol/L, and 60% of the young MI population with both a family history of premature CAD and LDL >4.14 mmol/L. FH has a morbidity of 1/313 in the general population, 1/31 in CAD patients, 1/15 in premature CAD patients, and 1/14 in hyperlipidemia patients, according to a meta-analysis published in 2020. Screening for FH and appropriate intervention are therefore an urgent priority. Although elevated LDL-C and early-onset CAD may improve the diagnostic efficiency of FH, a single biochemical screening test is insufficient to confirm the presence of FH because blood cholesterol levels vary with age, race, drug use, and pathological and physiological conditions; these factors frequently result in false-negative or false-positive diagnoses. Due to the overlap of LDL-C levels between FH and non-FH people, roughly 20% of members with *LDLR* mutations and modestly increased LDL-C levels go undetected when examined at the LDL-C level alone. Genetic testing is the “gold standard” for FH diagnosis, as shown in the recently released Expert Panel, and should be used to increase diagnostic rates. Chinese researchers believe that combining gene sequencing and DLCN criteria could improve the FH diagnosis rate in people with early MI and that all CAD patients under the age of 35 should be eligible for genetic testing because younger patients may benefit the most from early detection and statin therapy (Chen et al., 2019).

Over the last decade, genetic testing has advanced significantly and has become an important part of the clinical treatment of hereditary heart disease. It can reveal not just the illness’s underlying causes but also the disease risk in asymptomatic, high-risk family members (Ingles et al., 2020). On the one hand, genetic testing aids in the identification of FH, allowing for the initiation of rigorous lipid-lowering medication as soon as feasible. In a cohort of 469 Spaniards, genetic screening resulted in a 2.3-fold increase in FH detection rates (Sabatel-Perez et al., 2021). By studying the probable genetic origins of FH, genetic testing, on the other hand, gives prognostic information and enhanced risk stratification for FH (Aparicio et al., 2023). At the same LDL-C level, genetic abnormalities dramatically increase the risk of CAD. Patients with monogenic FH show a much higher risk of cardiovascular disease than those with polygenic FH and therefore have a greater need for lipid-lowering therapy. Improved diagnosis of FH, beginning or continuation of treatment, adjustment of medication and improvement of total or LDL cholesterol levels are all clinical and other qualitative results of FH genetic testing. In this work, we present enough genetic evidence to demonstrate that the insertion mutation in *LDLR* that results in hyperlipidaemia. We initially discovered a clinical patient from this family with a phenotype similar to HeFH. He inherited the mutation from his mother and therefore the clinical signs of typical FH were observed in this patient. Due to delayed illness identification and medication therapy, this patient has acquired significant atherosclerosis, but thankfully has not yet turned into cardiovascular adverse events. Later in life, interventions to control LDL-C levels are necessary to lower the likelihood of cardiovascular events. According to review of the literature, the majority of HeFH patients do not experience symptoms until they are adults, between the third and seventh decades of their life. Other patients in this family line are also HeFH patients carrying a mutation that may have been inherited autosomally dominantly from either of their parents. Typically, HeFH individuals have a 2/3 decrease in LDL clearance, which leads to an increase in circulating LDL-C of 1-2 fold (5–10 mmol/L; 200–400 mg/L). Genetic testing is crucial in this family and in the population where FH is widespread since persistent cholesterol deposition has been shown to cause atherosclerotic damage and even malignant cardiovascular catastrophes. And, as has been demonstrated, chronic cholesterol deposition can induce atherosclerotic damage and even major adverse cardiovascular events, so genetic testing in this family and in the FH-prevalent population is essential.

In contrast to medication, leading a healthy lifestyle is always the best way to treat FH. The risk factors for FH can be completely eliminated by quitting smoking, eating a healthy diet, exercising frequently, and maintaining a normal weight. Statins are the first-line treatment for FH, which are a family of medications that lower cholesterol production by inhibiting hydroxymethylglutaryl-CoA reductase, the rate-limiting enzyme in lipid synthesis. Statins have been frequently recommended for the treatment of hypercholesterolemia since their introduction in 1987. Statins include atorvastatin, fluvastatin, lovastatin, pitavastatin, pravastatin, rosuvastatin, and simvastatin, with atorvastatin being the most frequently used. Despite the fact that statins have a very favorable risk-benefit ratio, there are issues with medication intolerance and the “rule of 6%” (each doubling of the statin dose reduces LDL-C levels by only about 6% extra). Doctors must consult with patients to regularly check lipid levels in order to improve patient compliance and achieve long-term stable control of lipids. Whereas HeFH patients maintain half of their LDLR activity and react well to statins, HoFH patients do not since they have lost their LDLR function. Recent advances in molecular and cellular biology have facilitated the emergence of various novel lipid-lowering drugs. Hence, the focus on lipid-lowering medications has switched away from statins and toward PCSK9 inhibitors. PCSK9 is a serine protease that binds to LDLR and promotes its degradation via the lysosomal route; blocking PCSK9 limits LDLR degradation increasing LDLR availability and subsequently enhancing LDL clearance (Horton et al., 2009). These two monoclonal antibodies, Repatha (Evolocumab) and Praluent (Alirocumab) are the most commonly used PCSK9 inhibitors. Evolocumab decreased LDL-C by roughly 60% (from 92 mg/dL to 30 mg/dL, P < 0.001) and observably reduced the incidence of cardiovascular events by 15% in the Further Cardiovascular Outcomes Research with PCSK9 Inhibition in Subjects with Elevated Risk (FOURIER) research (Sabatine et al., 2017; Koren et al., 2019). Similarly, Alirocumab decreased LDL-C levels by 62.7% (37.6 mg/dL vs. 93.3 mg/dL) at week 16 in the ODYSSEY trial, reducing the probability of major adverse cardiovascular events (MACE) (Szarek et al., 2019). The latest research found that a novel fully human PCSK9 monoclonal antibody, Tafolecimab, yielded signifcant and persistent reductions in LDL-C levels and showed a favorable safety profle in Chinese patients with HeFH (Chai et al., 2023). Repatha and Praluent are now the market leaders, but a new generation of therapeutic siRNA is on the way. According to the rule of biological drug development, the next-generation of siRNA medications is destined to succeed (Ray et al., 2018).

Drug reactions in FH patients are quite variable. Patients’ phenotype and responsiveness to lipid-lowering medication in FH patients can be affected by the type of *LDLR* mutations they possess. *LDLR* variations are classified as null variants or defect variants according to ACMG criteria. Null variations are classified as nonsense, splicing, displaced deletion/insertion, and large rearrangement, whereas faulty mutations are defined as missense, non-displaced deletion/insertion, and promoter variation (Richards et al., 2015). Null variant carriers are said to have greater LDL levels, and rates of early CAD than faulty carriers, and null variants have also been linked to a poor response to statin therapy (Di Taranto et al., 2020). The number of alleles, in addition to the kind of variations, influenced the therapeutic impact. For example, treating HeFH and HoFH patients with PCSK9 inhibitors might reduce LDL-C by 56% and 38%, respectively (Galema-Boers et al., 2017). As a result, functionally characterization and classification of *LDLR* variations can be utilized to pick more tailored lipid-lowering medications and dyslipidemia management techniques, potentially improving patient results.

In our situation, the proband was diagnosed with dyslipidemia during a regular physical examination in 2015 (at the age of 42), with LDL-C levels up to 7.86 mmol/L and total cholesterol levels up to 9.9 mmol/L. This health assessment, however, did not draw the patient’s attention owing to a lack of awareness, and no additional examination or treatment was performed. Physical checks over the next 2 years revealed that his LDL-C values remained elevated. However, no more steps have been made. The patient was examined again at the end of 2017, and a coronary CTA test revealed coronary atherosclerosis with triple vessel lesions. This examination drew the patient’s attention, and he began taking aspirin and atorvastatin under the doctor’s supervision. After 1 month of therapy, LDL-C and total cholesterol levels dropped to 4.03 mmol/L and 5.59 mmol/L, respectively. The patient was prescribed statins, ezetimibe, and a PCSK9 inhibitor every 2 weeks for the following 3 years. Now, with good physical condition and no cardiovascular problems over this time, the level of LDL-C is properly regulated. At our recommendation, the patient agreed to undertake genetic testing. Genetic testing revealed a novel mutation in a known pathogenic gene (LDLR: c.2517_2518insCA) of FH in this case. The mutation was found as the cause of FH in the patient after several analyses. Some researchers thoroughly studied and evaluated LDLR variants in the Chinese population, discovering that the majority of *LDLR* variants in Chinese people were discovered in exon 4, with about 60% of missense mutations (Jiang et al., 2015). Furthermore, the pathogenicity of just a few *LDLR* mutations has been shown. Our newly found mutation was detected in exon 17 and was characterized as a frameshift insertion mutation, commonly known as a null variation. Our findings expand the range of HF variations in the Chinese population. Further cascade testing of family members revealed three carriers of the mutation, all of whom were young adults, one of whom had hyperlipidemia, and two younger kids who had no dyslipidemia. After age is taken into account, these three carriers require strict monitoring, and lipid changes should be monitored on a regular basis. Since the family has a heterozygous mutation, contemporary pharmacological treatments can target and treat this sort of mutation. If carriers receive early diagnosis and lipid-lowering medication, cardiovascular events can be avoided. As the research did not screen all members of the family for a number of reasons, it’s likely that some carriers went undiscovered. However, genetic testing for the *LDLR*, c.2517_2518insCA mutation in family members might help with early detection of FH patients, lifetime lipid-lowering medication, genetic counseling, and prenatal diagnosis. Because genetic screening plays a key role in the diagnosis and treatment of FH, this pedigree necessitates genetic testing. Patients who are at high risk of cardiovascular disease or who do not react well to lipid-lowering medication can be predicted and selected using genetic features. Genetic testing might aid in the early detection of FH, allowing for the initiation of rigorous lipid-lowering medication and the prevention of future cardiovascular events. Furthermore, persons who carry the pathogenic mutation have a higher risk of cardiovascular disease and require more severe therapy and follow-up.

In conclusion, genetic testing revealed a previously unknown harmful frameshift insertion mutation in LDLR (c.2517_2518insCA) from an FH family. This LOF mutation is considered to affect RNA stability via altering the RNA molecule’s free energy dynamics, as well as modifying protein structure by eliminating an important LDLR functional region. Further cascade screening of family members discovered three carriers of the mutation, which require thorough monitoring of lipid changes and intensive follow-up in order to avoid future cardiovascular problems. Finally, we stress the significance of genetic testing and screening for all family instances of FH in order to learn more about the disease’s genetic and clinical manifestations. In addition, the development of personalized drug therapy for carriers of different mutations is critical for prevention and prognosis of FH.

## Data Availability

The raw sequence data reported in this paper have been deposited in the Genome Sequence Archive (Genomics, Proteomics & Bioinformatics 2025) in National Genomics Data Center (Nucleic Acids Res 2025), China National Center for Bioinformation / Beijing Institute of Genomics, Chinese Academy of Sciences (GSA-Human: HRA017682) that are publicly accessible at https://ngdc.cncb.ac.cn/gsa-human.
